# A new staging system for nasopharyngeal carcinoma based on intensity-modulated radiation therapy: results of a prospective multicentric clinical study

**DOI:** 10.18632/oncotarget.7553

**Published:** 2016-02-21

**Authors:** Min Kang, Jianxiong Long, Guisheng Li, Haolin Yan, Guosheng Feng, Meilian Liu, Jinxian Zhu, Rensheng Wang

**Affiliations:** ^1^ Department of Radiation Oncology, The First Affiliated Hospital of Guangxi Medical University, Nanning, Guangxi, P.R. China; ^2^ School of Public Health, Guangxi Medical University, Nanning, Guangxi, P.R. China; ^3^ Department of Radiation Oncology, Liuzhou Worker Hospital, Liuzhou, Guangxi, P.R. China; ^4^ Department of Radiation Oncology, First People's Hospital of Yulin City, Yulin, Guangxi, P.R. China; ^5^ Department of Radiation Oncology, People's Hospital of Guangxi Zhuang Autonomous Region, Nanning, Guangxi, P.R. China; ^6^ Department of Radiation Oncology, Affiliated Hospital of Guilin Medical University, Guilin, Guangxi, P.R. China; ^7^ Department of Radiation Oncology, Wuzhou Red Cross Hospital, Wuzhou, Guangxi, P.R. China

**Keywords:** nasopharyngeal carcinoma, intensity modulated radiation therapy, staging systerm, prognosis

## Abstract

**Purpose:**

To establish a new clinical staging standard for nasopharyngeal carcinoma (NPC), based on intensity-modulated radiotherapy (IMRT), through a prospective multicenter clinical trial.

**Experiment Design:**

492 NPC patients were selected from six hospitals in the Guangxi Zhuang Autonomous Region, China from January 2006 to December 2009. Kaplan-Meier method was adopted to calculate survival rates. Log-rank test was used to compare survival differences.

**Results:**

According to the seventh edition of the UICC/AJCC staging system, the differences between T1, T2 and T3 are not statistically significant, suggesting that T1, T2 and T3 could be combined as new T1. There were significant differences between all N stages except those of N3a and N3b, suggesting that N3a and N3b could be combined as new N3. Additionally, the overall survival (OS) curves of stages I, II, III and IVa were not significantly different. Therefore, we propose a new clinical NPC staging standard based on magnetic resonance imaging (MRI) and IMRT as T stage (including T1 and T2), N stage (including N0, N1, N2 and N3) and clinical staging includes I (T1N0M0), II (T1N1-2M0, T2N0M0), III (T2N1-2M0), IVa (TxN3M0) and IVb (TxNxM1). Recommended staging system performs better in risk difference and distribution balance. Furthermore, the differences in the 5-year curves of local relapse-free survival (LRFS), distant metastasis-free survival (DMFS), and OS were all statistically more significant than the seventh edition of the UICC/AJCC staging system.

**Conclusions:**

Proposed staging system is more adaptable to IMRT and predicts the prognosis of NPC patients more accurately.

## INTRODUCTION

Based on the principles of invasion area of lesion and progress, the TNM clinical staging system for NPC divides the severity of carcinomas (termed either “*in situ*” (Tumor, T), “regional lymph node metastasis” (Node, N) or “distant metastasis” (Metastasis, M)) into several levels, such as T1-4, N0-3 and M0-1. Informed by the prognosis of the three above mentioned degrees of severity, the NPC is divided into four stages, from I to IV stage. The T stage is relevant to the local control rate, while the N stage reflects the risk level of distant metastasis. The intent and significance of clinical staging is as follows: (1) to guide therapeutic planning; (2) to aid prognosis; (3) to evaluate curative effect; (4) to facilitate both information exchange and effect comparison between treatment entities; (5) and to support the continuity of research work on cancer [1]. It is self-evident that the TNM clinical staging system for NPC should be amended when the progress of diagnostic and therapeutic methods allows for finer distinctions or more accurate definitions.

In the past 10 years, NPC diagnostics and therapy have seen dramatic improvements. Firstly, MRI has become the preferred imaging method for detecting NPC. Extensive research has shown that compared to computed tomography (CT), MRI displays the invasion area of the lesions more clearly, which in turn allows for a change in staging [2, 3]. Secondly, IMRT has gradually replaced regular two-dimensional radiotherapy (2D-RT) as the mainstream therapy for NPC [4- 6]. Combination therapy, with IMRT as the primary therapy, has dramatically improved the prognosis of NPC patients, and the 5-year overall survival rate has reached 80% [7-8]. While significantly improving local control rates for carcinoma, this technology also reduces the side effects of radiotherapy [9]. However, the seventh edition of the benchmark staging volume Union for International Cancer Control/American Joint Committee on Cancer (UICC/AJCC), published in 2009, is based on data from regular 2D-RT and thus is not informed by innovations in diagnosis and therapy on staging [10]. A clinical staging standard adaptable to IMRT has not yet been formulated. It is a key task to establish such a standard to make prognosis more accurate and treatment more effective for NPC patients.

In this study, we performed a prospective multicenter clinical trial including 492 NPC patients to establish a new clinical staging standard for NPC based on IMRT.

## MATERIALS AND METHODS

### Clinical data

Four hundred and ninety-two NPC patients were selected from six hospitals in Guangxi Zhuang Autonomous Region, China from January 2006 to December 2009. 338 were male and 154 were female. The median age of the group was 45 years old (18-81). All the patients received IMRT. Patients with a Karnofsky performance status of 70 or more, who met criteria for blood counts and other tests (i.e., serum creatinine ≤ 1.6 mg/dl and serum bilirubin ≤ 1.5 mg/dl; white blood cell ≥ 3600/mm^3^, platelet ≥ 100,000/mm^3^, and hemoglobin ≥12.0 g/dl for male, ≥ 11.0 g/dl for female) were eligible. Before treatment, all patients underwent a detailed physical examination, a general situation appraisal, a routine blood examination, a nasopharyngeal fiberscope examination, and imaging, such as a chest X-ray or CT, abdominal ultrasound, MRI of nasopharynx and neck. Patients at N2-N3 stage received additional bone scanning. All patients were estaged with according to the criteria of the 7th edition for the current study. Prior written and informed consent was obtained from every patient and the study was approved by the Ethics Review Board of Guangxi Medical University.

### Clinical staging method

All patients' MRI scans were independently reviewed on the PACS system by two physicians. If their diagnoses were dissimilar, the research group defined the stage according to seventh edition of the UICC/AJCC clinical staging standard, incorporating information from patients' physical examinations, such as cranial nerve palsies and the size of lymph nodes. Lymph nodes were identified according to the 2013 edition of the Radiation Therapy Oncology Group (RTOG)'s identification guidelines for nodal classification in the neck.

### MRI scanning method

The MRI was performed with a GE Signa 1.5T Magnetic Resonance Scanner. All cases received conventional and enhanced scanning. Scanning patterns include cross section, sagittal section and coronal section scan T2WI (TR 3000~4000ms, TE102~110ms), T1WI (TR 2200~2400ms, TE77~109ms, TI 750ms) and T1WI enhanced scan with position and parameters the same as those of the T1WI plain scan. The quadrature head coil was adopted with slice thickness 6 mm, slice gap 1 mm and matrix 256 × 192. The cross section scan field ranged from the suprasellar cistern to the bottom edge of the clavicle. Gd-DTPA was used as the contrast agent.

### Therapeutic method

The four hundred and ninety-two NPC patients received IMRT throughout the entire process. Adopting the dorsal decubitus position, patients were fixed with a head-and-neck thermoplastic mask and placed under the CT simulated positioning system for the enhanced CT scan. The scan field ranged from the calvarium to 3 cm below the clavicle. The gaps and thicknesses of slices were both 3 mm.

Under the guidance of Report 50 and Report 62 of the International Commission on Radiation Units and Measurements (ICRU), delineation of tumor target volume was made by delineating the target area on CT film slice by slice with MRI plain scan plus enhanced scan. Gross tumor volume (GTV) included the primary site of the carcinoma and its invasion area (GTVnx), metastatic retropharyngeal lymph nodes (GTVrpn), and metastatic cervical lymph nodes that were visible *via* imaging method and clinical examination. The area of clinical target volume (CTV) was adjusted according to the involved situation. CTV1 included (GTVnx + GTVrpn) + 5~10mm; CTV2 covered CTV1. Additionally, according to the specific carcinoma invasion region and its area, it was necessary to consider whether to include locations such as the postnaris, pterygopalatine fossa, post-maxillary sinus, part of the posterior ethmoidal sinus, the parapharyngeal space, the base of the skull, part of the cervical vertebra and the Basilar clivus. The planning target volume (PTV) included allowances for set-up error and organ movement during the treatment process and was generally extended by 3~5 mm on the basis of GTVs and CTVs. Organs at risk (OAR) were defined as the spinal cord, brain stem, temporal lobe, hypophysis, optic nerve, optic chiasm, optic lens, eyeball, and parotid gland. Prescribed doses were delivered as follows: 68~74Gy for PGTVnx and PTVrpn; 66~70Gy for PTVnd; 60~64Gy for PTV1; 50~56Gy for PTV2; 5 times/week; 30~33 times in total. According to RTOG 0225 protocols, dose restrictions for organs at risk were set as follows: lens ≤ 8Gy; parotid gland D33 ≤ 35Gy; eardrum ≤ 50Gy; brain stem ≤ 54Gy; optic nerve ≤ 54Gy; optic chiasm ≤ 54Gy; hypophysis ≤ 54Gy; spinal cord ≤ 45Gy; temporal lobe ≤ 60Gy; mandibular ≤ 60Gy; temporomandibular joint ≤ 60Gy; and as low an exposure dose as possible for the oral cavity.

Primary sites and the neck were irradiated with coplanar radiation fields. The dose distribution on the target volumes and organs at risk was appraised slice by slice according to dose volume histogram (DVH) and CT film. PTV required that the volume percentage relevant to 100% prescription dose curve be equal to or greater than 95%; the PTV volume percentage relevant to not less than a 110% prescription dose curve be less than 20%; the PTV volume percentage relevant to not less than a 115% prescription dose curve be less than 5%; and the PTV volume percentage relevant to less than a 93% prescription dose curve be less than 1%.

All stages were defined according to the seventh edition of the UICC/AJCC staging standards, Of the 477 patients with Stage II-IVB disease, 93.70%(461/492)received platinum-based chemotherapy. All centers used identical chemotherapy protocols. Of these cases, 51.0% (235/461) received concurrent chemotherapy; 37.09% (171/461) received induction-concurrent chemotherapy; 7.59% (35/461) received concurrent-adjuvant chemotherapy; 4.12% (19/461) received induction-concurrent-adjuvant chemotherapy; and 0.22% (1/461) received induction chemotherapy.

### Random follow-ups

Random follow-ups began 3 months after end of treatment. The follow-up period was defined as starting from the date of commencement of treatment to the date of the last random follow-up, or to the time of death. By December 31, 2014, the date of the last random follow-up, the median follow-up period was 64.1 months (6~92 months). A percentage of 96.3% had complete follow-up data for 5 years. Major analytic indexes included overall survival (OS), disease-free survival (DFS), local relapse-free survival (LRFS), nodal relapse-free survival (NRFS) and distant metastasis-free survival (DMFS).

### Statistical method

SPSS18.0 software was used for statistical analysis. Kappa analysis, Kaplan-Meier method and Long-rank test were used respectively, to compare the distribution consistency of cases at different stages, to calculate all kinds of survival rates and to interrogate differences in survival rates. *P* < 0.05 was deemed statistically significant.

## RESULTS

### Patients' stages and treatment effect

According to the seventh edition UICC/AJCC staging standards, the proportions of I, II, III, IVa, and IVb were 3.0% (15/492), 14.4% (71/492), 35.8% (176/492), 38.0% (187/492), and 8.7% (43/492), respectively; the proportions of T1, T2, T3, and T4 were 6.7% (33/492), 18.5% (91/492), 33.7% (166/492), and 41.1% (202/492), respectively; the proportions of N0, N1, N2, N3a, and N3b were 13.0% (64/492), 32.3% (159/492), 45.9% (226/492), 2.2% (11/492), and 6.5% (32/492), respectively (Table 1). The 5-year overall survival (OS), disease-free survival (DFS), relapse-free survival (RFS), and distant metastasis-free survival (DMFS) rates for the whole group were 80.5%, 78.6%, 94.1%, and 84.3%, respectively.

**Table 1:** The distribution of T, N stage of 492 nasopharyngeal carcinoma patients (UICC2010 staging systems)

Stage	N0	N1	N2	N3a	N3b	Total
T1	14	16	3	0	0	33
T2	25	31	24	4	7	91
T3	15	64	70	1	16	166
T4	10	48	129	6	9	202
Total	64	159	226	11	32	492

### Comparison of LRFS curves of T stage, DMFS curves of N stage and OS curves of clinical stage (UICC/AJCC staging standard)

**Figure 1:**
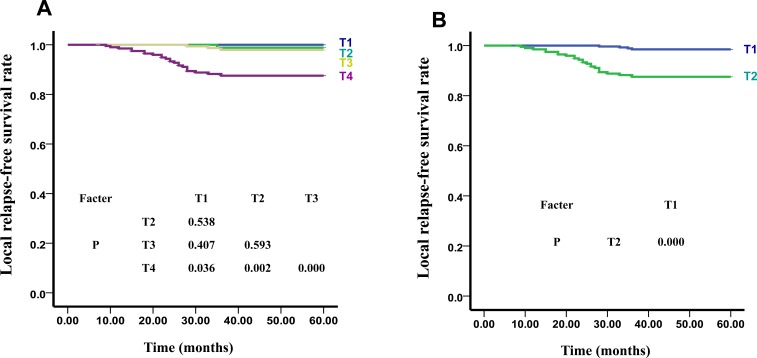
The LRFS Curves of T stage of UICC **A.** and our New Recommended staging systems **B.**

According to UICC/AJCC staging standards, the 5-year LRFS curves of T1, T2, T3, and T4 were 100%, 98.2%, 97.9%, and 88.2%, respectively (x^2^ = 25.916, *P* < 0.01). The differences of the LRFS curves of T1 and T2 (*x*^2^ = 0.379, *P* > 0.05), T1 and T3 (*x*^2^ = 0.687, *P* > 0.05), T2 and T3 (*x*^2^ = 0.285, *P* > 0.05) were not statistically significant. However, the differences in the LRFS curves between T4 and the other 3 stages were statistically significant: T1 and T4 (*x*^2^ = 4.381, *P* < 0.05); T2 and T4 (*x*^2^ = 9.629, *P* < 0.01); T3 and T4 (*x*^2^ = 13.759, *P* < 0.01) (Figure 1A). LRFS and OS comparisons among the T categories are shown in Table 2. Hazard ratios (HRs) for LRFS and OS between T1 and T2, and between T2 and T3 did not differed significantly (*P* > 0.05). T4 had significantly higher hazard risk of both LRFS and OS failures compared with other T stages (HR = 7.031 and 3.441, respectively, *P* < 0.01) (Table 2).

**Table 2:** Independent significance of T and N-categories by multivariate analyses of 492 nasopharyngeal carcinoma patients

Failure	Category	comparison	Dealth	
			HR (95% CI)	*P* value
5-yr OS	T1			
	T2	T2 *vs* T1	2.703(0.333-21.979)	0.352
	T3	T3 *vs* T2	1.081(0.703-1.661)	0.722
	T4	T4 *vs* T3	3.441(1.536-7.712)	0.003
	T1+T2+T3	T1 +T2 +T3 *vs* T4	1.657(1.101-2.495)	0.016
	N0			
	N1	N1 *vs* N0	1.592(1.208-2.098)	0.001
	N2	N2 *vs* N1	2.458(1.432-4.219)	0.001
	N3a	N3a *vs* N2	2.857(1.225-6.662)	0.015
	N3b	N3b *vs* N3a	0.847(0.329-2.184)	0.731
	N3a+ N3b	N3a+ N3b *vs* N2	2.507(1.508-4.169)	0.000
5-yr LRFS	T1			
	T2	T2 *vs* T1	3.339(0.000-3.926)	0.712
	T3	T3 *vs* T2	1.835(0.191-17.653)	0.599
	T4	T4 *vs* T3	7.031(2.11-23.422)	0.001
	T1+T2+T3	T1 +T2 +T3 *vs* T4	9.445(3.265-27.324)	0.000
5-yr DMFS	N0			
	N1	N1 *vs* N0	1.750(1.310-2.338)	0.000
	N2	N2 *vs* N1	2.116(1.170-3.824)	0.014
	N3a	N3a *vs* N2	3.534(1.499-8.332)	0.004
	N3b	N3b *vs* N3a	0.821(0.315-2.140)	0.687
	N3a+ N3b	N3a+ N3b *vs* N2	2.758(2.012-3.780)	0.000

**Figure 2:**
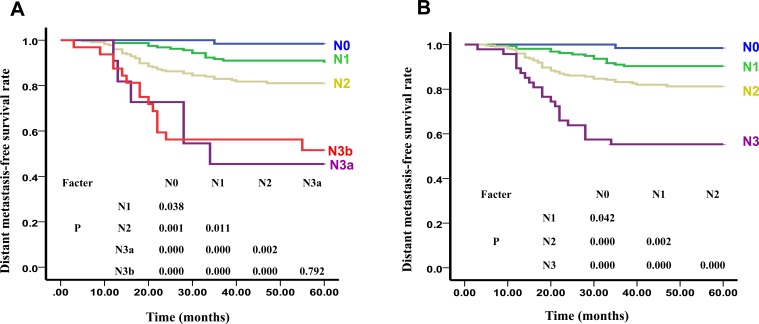
The DMFS Curves of N stage of UICC **A.** and our New Recommended staging systems **B.**

As shown in Figure 2A, the 5-year DMFS curves of N0, N1, N2, N3a, and N3b were 98.4%, 90.4%, 81.1%, 45.5%, and 49.3% (x^2^ = 57.489, *P* < 0.01), respectively. The difference between N3a and N3b (*x*^2^ = 1.141, *P* > 0.05) was not statistically significant. However, the differences between N0 and N1 (*x*^2^ = 4.325, *P* < 0.05), N0 and N2 (*x*^2^ = 11.197, *P* < 0.01), N0 and N3a (*x*^2^ = 35.224, *P* < 0.01), N0 and N3b (*x*^2^ = 31.973, *P* < 0.01), N1 and N2 (*x*^2^ = 6.491, *P* < 0.05), N1 and N3a (*x*^2^ = 22.421, *P* < 0.01), N1 and N3b (*x*^2^ = 31.427, *P* < 0.01), N2 and N3a (*x*^2^ = 9.797, *P* < 0.01), and N2 and N3b (*x*^2^ = 12.721, *P* < 0.01) were all statistically significant. HR for DMFS and OS between the N0 and N1, N1 and N2, and N2 and N3a differed significantly, but this was not the case between N3a and N3b. When combining N3a and N3b, the differences in HRs for both DMFS and OS were significant (HR = 2.758 and 2.507, respectively, *P* < 0.01).

**Figure 3:**
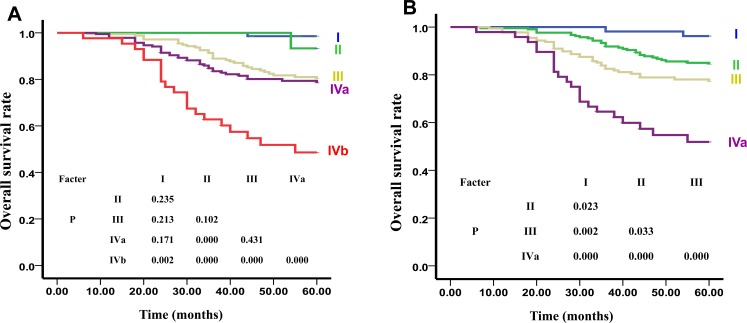
The OS Curve of Clinical Stages of UICC **A.** and our New Recommended staging systems **B.**.

As shown in Figure 3A, the 5-year OS curves of Clinical I, II, III, IVa, and IVb were 98.6%, 93.3%, 81.0%, 79.4%, and 45.5% (x^2^ = 54.040, *P* < 0.01), respectively. The differences between I and II (*x*^2^ = 1.408, *P* > 0.05), I and III (*x*^2^ = 1.551, *P* > 0.05), I and IVa (*x*^2^ = 1.878, *P* > 0.05), II and III (*x*^2^ = 1.44, *P* > 0.05), III and IVa (*x*^2^ = 0.619, *P* > 0.05) were not statistically significant. However, the differences between I and IVb (*x*^2^ = 8.481, *P* < 0.01), II and IVa (*x*^2^ = 14.652, *P* < 0.01), II and IVb (*x*^2^ = 41.158, *P* < 0.01), III and IVb (*x*^2^ = 27.433, *P* < 0.01), and also between IVa and IVb (*x*^2^ = 18.017, *P* < 0.01) were all statistically significant. HR for OS were significantly different only in when comparing of stage IVb and IVc with stage I (*P* < 0.05), and not when stages II, III, and IVa were compared with stage I (*P* > 0.05) (Table 4).

### New recommended staging standard

The above results of LRFS curves of NPC patients showed that there was a statistically significant difference only between T4 and the other 3 stages, which, we suggest, means that T1, T2, and T3 can be combined to form T1 in a new staging system. In addition, the DMFS curves of N3a and N3b were similar, thus N3a and N3b can also be combined as N3 in a new staging system. The prognosis of NPC patients with distant metastases was usually poor. Therefore, we suggest dividing stage IV into IVa and IVb according to our data. Our suggested new clinical NPC staging standard based on MRI and adaptable to IMRT is, then, as follows: T stage (including T1 and T2) and N stage (including N0, N1, N2, and N3). Our suggested clinical staging includes I (T1N0M0), II (T1N1-2M0, T2N0M0), III (T2N1-2M0), IVa (TxN3M0) and IVb (TxNxM1).

### Appraisal of the new recommended staging standard survival predictive value

As shown in Figure 1B, there were significant differences between the 5-year LRFS curves of sub-stages in our new recommended T stage: T1:T2 (*x*^2^ = 23.723, *P* = 0.000). The differences between the 5-year DMFS curves of sub-stages in our new recommended N stage were also found to be statistically significant: N0:N1 (*x*^2^ = 4.119, *P* < 0.05), N0:N2 (*x*^2^ = 13.537, *P* < 0.01), N0:N3 (*x*^2^ = 34.401, *P* < 0.01), N1:N2 (*x*^2^ = 9.917, *P* < 0.01), N1:N3 (*x*^2^ = 38.127, *P* < 0.01), N2:N3 (*x*^2^ = 13.594, *P* < 0.01) (Figure 2B). Additionally, statistically significant differences were noted between the 5-year OS curves of our new recommended clinical stages: I:II (*x*^2^ = 5.172, *P* < 0.05), I:III (*x*^2^ = 9.950, *P* < 0.01), I:IVa (*x*^2^ = 28.115, *P* < 0.01), II:III (*x*^2^ = 4.547, *P* < 0.05), II:IVa (*x*^2^ = 32.179, *P* < 0.01), III:IVa (*x*^2^ = 12.070, *P* < 0.01) (Figure 3B).

### Distribution balance

The cases number and ratio of our new recommended staging system and also those of the UICC/AJCC staging system are listed in Table 3. In the UICC/AJCC staging system, the proportions of each T stage were T1 (6.7%), T2 (18.5%), T3 (33.7%), and T4 (41.1%), while in our new recommended staging system T1 and T2 are 58.9% and 41.1% respectively. The case distributions of the N stages in both staging systems were equivalent. Turning to the comparative distribution of the clinical stages, the numbers in the I and II stages of the new system we propose were greater than those of the UICC/AJCC system (10.3%, 40.5% *vs*. 2.9%, 13.5%, respectively). Overall, the distribution balance of our proposed new staging system is better than that of UICC staging system.

**Table 3:** Comparison of distribution balance between two staging systems cases (%)

stage		UICC system	Proposed system
T stage			
T1		33 (6.7)	290(58.9)
T2		91(18.5)	202(41.1)
T3		166(33.7)	-
T4		202(41.1)	-
N stage			
N0		64(13.0)	64(13.0)
N1		159(32.3)	159(32.3)
N2		226(45.9)	226(45.9)
N3	N3a	11(2.2)	43(8.7)
	N3b	32(6.5)	
M stage			
M		34 (6.5)	34 (6.5)
Clinical stage			
I		15(2.9)	54(10.3)
II		71(13.5)	213(40.5)
III		176(33.5)	177(33.7)
IVa		187(35.6)	48(9.1)
IVb		43(8.2)	34 (6.5)
IVc		34(6.5)	-

### Risk difference

The overall survival hazard ratios between our proposed new staging system and that of the UICC/AJCC are set out in Table 4, where stage I is chosen as the benchmark (HR = 1). The risk differentials between each stage of our proposed system are all higher than those of the UICC/AJCC staging system. Moreover, we found significant risk differentials when comparing stage I with other stages in our system, where in the UICC/AJCC staging system they are to be found only when comparing stages IVb and IVc with stage I. We also found that the risk at stage IVb of our proposed system was dramatically higher than that at stage IVc of the UICC/AJCC staging system (91.642 *vs*. 51.297), even though both consist of M1 patients.

**Table 4:** Comparison of risk difference between two staging systems

N stage	N	Overall survival Hazard ratio(95% CI)
Proposed system		
I	54(10.3)	1
II	213(40.5)	4.151(1.058-18.494)*
III	177(33.7)	7.382(1.777-30.658)*
IVa	48(9.1)	19.508(4.576-83.167)*
IVb	34 (6.5)	91.642 (21.198-396.190)*
UICC system		
I	15(2.9)	1
II	71(13.5)	1.311(0.358-4.810)
III	176(33.5)	3.165(0.432-23.171)
IVa	187(35.6)	3.824(0.524-27.883)
IVb	43(8.2)	11.996(1.611-89.351)*
IVc	34(6.5)	51.297(6.833-385.106)*

* *P* < 0.05

## DISCUSSION

In the past 10 years, diagnosis and therapy for NPC have dramatically improved. Intensity-modulated radiotherapy (IMRT) has gradually replaced two-dimensional radiotherapy (2D-RT) as the mainstream therapy for NPC [11]. Combination therapy, with IMRT as the lead therapy, has dramatically improved the prognosis of NPC patients and the 5-year overall survival has reached 80% [12-17]. Zong et al. [18] surveyed 1241 nasopharyngeal carcinoma (NPC) patients treated with IMRT, of whom 88.7% received concurrent platinum-based chemotherapy. The rates of 5-year OS, DSS, DMFS, RRFS, and LRFS were 81.1%, 82.6%, 82.6%, 95.4%, and 92.9% respectively. In the present study, the 5-year overall survival (OS), disease-free survival (DFS), relapse-free survival (RFS), and distant metastasis-free survival (DMSF) of the entire cohort are 80.5%, 78.6%, 94.1%, and 84.3%, respectively. This is consistent with other reports.

The TNM staging system is a comprehensive and inclusive system incorporating a host of prognostic factors confirmed by research in clinical epidemiology, and identifying new prognosis factors depends on improvements in diagnosis and therapy, which, because they are ongoing and continuous mean that due to prognosis factors are also changing. This in turn requires the, staging system to be regularly updated. The seventh edition of the UICC/AJCC staging standard the published in 2009 is largely based on the data and results of regular two-dimensional radiotherapy. IMRT, which is more targeted and more accurate than the old two-dimensional method, has been applied more and more frequently in the treatment of NPC. A number of studies have shown that the survival rates between each T stage in nasopharyngeal carcinoma patients were not significantly difference between each T stage after treatment with IMRT [16, 17, 19]. Han, et al. conducted a retrospective analysis on 305 patients who had received IMRT and found that the T stage was no longer the most influential factor in the local regional control rate and the overall survival rate [20]. Lin, et al. have also indicated that the T stage was no longer the factor that most influences outcomes in clinical treatment, but that the N stage was the key prognostic factor influencing distant metastasis-free survival and overall survival rates [9]. The results of one survey group indicated that the differences in local relapse risk ratios between T2b and T1, T2b and T2a, and T2b and T3 were not statistically significant [19]. Studies have showned that differences in overall survival between T1 and T2a, T2b sub-stages were not statistically significant [21-23], and the prognosis of T2N0 and T1N1 of stage II were similar to that at T1N0 of stage I [23-24]. Additionally there is some disagreement over the utility of having maximum lymph node diameter as the independent prognostic factor, identified through palpation in the N stage of NPC [19, 21 23, 25, 29]. In this study, we observed 492 NPC patients with initial treatment and analyzed the relationship between prognosis and staging. According to the seventh edition of the UICC/AJCC staging system, the differences between T1, T2, and T3 were not statistically significant, while those between T4 and the other 3 stages were all statistically significant. Moreover, there were significant differences between each stage except N3a and N3b. Further, the OS curves of stage I, II, III, and IVa were not significantly different other between each other. These results indicated that the seventh edition of the UICC/AJCC staging system is inappropriate for the staging of patients who have received IMRT.

Lee [30] found, in a study of 985 NPC patients treated with 3D conformal radiotherapy or IMRT, that the difference in LRFS between T2 and T3 was significant (*P* = 0.043), but not that the difference between the LRFS of T1 and T2 (*P* = 0.99). Zong et al [18], meanwhile, performed a retrospective analysis of 1241 newly diagnosed NPC patients without distant metastasis who received IMRT. All MRIs were independently reevaluated and restaged according to the 7th edition UICC/AJCC staging system, and the relationship between prognostic factors and staging were analyzed. They found that the hazard ratios (HRs) for DSS and OS between T2 and T3 as well as between T3 and T4 differed significantly, but not those between T1 and T2. Therefore, they suggested combining T1 and T2 into T1, and changing T3 and T4 to T2 and T3, respectively. Chen et al.[31] conducted a retrospective study of 512 NPC patients treated with IMRT, and reported that the LRFS and DFS of T1 and T2 subjects, calculated using the 7th edition system were significantly different (*P* = 0.019 and *P* = 0.009). However, they were not between T2 and T3 (*P* = 0.874 and *P* = 0.589) [31]. These reports indicate that local control differences between T1-3 patients will be diminished when treated with 3DCRT or IMRT. In the present study, hazard ratios (HRs) for LRFS and OS between T1 and T2 and between T2 and T3 were not significantly different (*P* > 0.05). T4 subjects had a significantly higher risk of both LRFS and OS failures compared with other T stages (HR = 7.031 and 3.441, respectively, *P* < 0.01)(Table 2), suggesting that T1, T2, and T3 can be combined as T1 (T1 + T2 + T3 *vs* T4, HR = 9.445 and 1.657, respectively, *P* < 0.05).

Zong [18] found, in a retrospective analysis of 1241 newly diagnosed NPC patients without distant metastases who received IMRT, that the differences in DMFS between N0 and N1 and N1 and N2 were significant. However, no significant difference was found in DMFS between N2 and N3a, or between N2 and N3b. HR for DSS and OS between N0 and N1,and between N1 and N2, differed significantly, but not between N2 and N3a, or N3a and N3b. In this study, there were significant differences in DMFS and OS between each stage except N3a and N3b. The differences in HR for DMFS and OS between N0 and N1, N1 and N2, and N2 and N3a were significant, but not between N3a and N3b. When combining N3a and N3b, the differences in HRs for both DMFS and OS were found to be significant (HR = 2.758 and 2.507, respectively, *P* < 0.01)(Table 2). Thus we suggest that N3a and N3b could be combined as N3. This notion is consistent with other studies.

In this study, we performed a multi-center prospective clinical trial with a cohort of 492 NPC patients presenting for initial treatment and analyzed the relationship between prognosis and staging. Regardless of the limitation of the number of cases, the results indicated that the 7th edition of the UICC/AJCC staging system still showed superior prognostic value in NPC patients treated with IMRT. Therefore, we propose a new clinical NPC staging standard based on MRI and adaptable to IMRT as follows: T stage (including T1 and T2) and N stage (incorporating N0, N1, N2, and N3). Our adjusted clinical staging is thus: I (T1N0M0), II (T1N1-2M0, T2N0M0), III (T2N1-2M0), IVa (TxN3M0), and IVb (TxNxM1). Compared to the seventh edition of the UICC/AJCC staging system, our proposed staging performs better in risk difference and distribution balance calculations. Furthermore, the differences between the sub-stages in the 5-year curves of LRFS, DMFS, and OS were all statistically significant in our adjusted system. We conclude that our proposed new staging system is better tuned to NPC patients undergoing IMRT and can predict their prognosis more accurately.
